# Road Traffic and Railway Noise Exposures and Adiposity in Adults: A Cross-Sectional Analysis of the Danish Diet, Cancer, and Health Cohort

**DOI:** 10.1289/ehp.1409052

**Published:** 2015-08-04

**Authors:** Jeppe Schultz Christensen, Ole Raaschou-Nielsen, Anne Tjønneland, Kim Overvad, Rikke B. Nordsborg, Matthias Ketzel, Thorkild IA Sørensen, Mette Sørensen

**Affiliations:** 1 Diet, Genes and Environment, Danish Cancer Society Research Center, Copenhagen, Denmark; 2 Department of Environmental Science, Aarhus University, Roskilde, Denmark; 3 Department of Public Health, Section for Epidemiology, Aarhus University, Aarhus, Denmark; 4 Department of Cardiology, Aalborg University Hospital, Aalborg, Denmark; 5 Novo Nordisk Foundation Center for Basic Metabolic Research and Department of Public Health, Faculty of Health and Medical Sciences, Copenhagen, Denmark; 6 Institute of Preventive Medicine, Bispebjerg and Frederiksberg Hospitals, The Capital Region, Copenhagen, Denmark; 7 MRC Integrative Epidemiology Unit, Bristol University, Bristol, United Kingdom

## Abstract

**Background:**

Traffic noise has been associated with cardiovascular and metabolic disorders. Potential modes of action are through stress and sleep disturbance, which may lead to endocrine dysregulation and overweight.

**Objectives:**

We aimed to investigate the relationship between residential traffic and railway noise and adiposity.

**Methods:**

In this cross-sectional study of 57,053 middle-aged people, height, weight, waist circumference, and bioelectrical impedance were measured at enrollment (1993–1997). Body mass index (BMI), body fat mass index (BFMI), and lean body mass index (LBMI) were calculated. Residential exposure to road and railway traffic noise exposure was calculated using the Nordic prediction method. Associations between traffic noise and anthropometric measures at enrollment were analyzed using general linear models and logistic regression adjusted for demographic and lifestyle factors.

**Results:**

Linear regression models adjusted for age, sex, and socioeconomic factors showed that 5-year mean road traffic noise exposure preceding enrollment was associated with a 0.35-cm wider waist circumference (95% CI: 0.21, 0.50) and a 0.18-point higher BMI (95% CI: 0.12, 0.23) per 10 dB. Small, significant increases were also found for BFMI and LBMI. All associations followed linear exposure–response relationships. Exposure to railway noise was not linearly associated with adiposity measures. However, exposure > 60 dB was associated with a 0.71-cm wider waist circumference (95% CI: 0.23, 1.19) and a 0.19-point higher BMI (95% CI: 0.0072, 0.37) compared with unexposed participants (0–20 dB).

**Conclusions:**

The present study finds positive associations between residential exposure to road traffic and railway noise and adiposity.

**Citation:**

Christensen JS, Raaschou-Nielsen O, Tjønneland A, Overvad K, Nordsborg RB, Ketzel M, Sørensen TI, Sørensen M. 2016. Road traffic and railway noise exposures and adiposity in adults: a cross-sectional analysis of the Danish Diet, Cancer, and Health cohort. Environ Health Perspect 124:329–335; http://dx.doi.org/10.1289/ehp.1409052

## Introduction

Traffic noise has been related to adverse health effects including cardiovascular disease (Munzel et al. 2014; Sørensen et al. 2011) and metabolic disorders (Sørensen et al. 2013), and a recent study found aircraft noise to be associated with an increase in waist circumference (Eriksson et al. 2014). A proposed mode of action is through a stress response with activation of the hypothalamus–pituitary–adrenal (HPA) axis leading to increased levels of noradrenaline, adrenaline, and corticosteroids, and activation of the sympathetic autonomic nervous system (Babisch 2003; Maschke et al. 2000). This mode of action is supported by studies indicating that exposure to traffic noise is associated with higher levels of cortisol and adrenalin (Griefahn and Robens 2010; Schmidt et al. 2013; Selander et al. 2009). Chronically high levels of cortisol may lead to alterations in the metabolism (Björntorp and Rosmond 2000), and it has been suggested by some studies that stress-induced alterations in cortisol level are related to metabolic changes including weight gain (Rosmond et al. 1998; Vicennati et al. 2009). However, the overall role of cortisol in the development of adiposity is not clear because findings in recent experimental studies have been inconsistent (Abraham et al. 2013).

Traffic noise is known to disturb sleep (Miedema and Vos 2007), and in experimental human studies noise has been associated with higher body motility, sleep state changes, sleep onset latency, nocturnal awakenings, and early morning awakenings (Pirrera et al. 2010). Furthermore, total sleep deprivation has been associated with increased food intake in both animals and humans (Nedeltcheva et al. 2009; Rechtschaffen and Bergmann 1995). The underlying mechanism may be dysregulation of “hunger-hormones” because the levels of the anorexigenic hormone leptin have been negatively associated with sleep deprivation (Chaput et al. 2007; Spiegel et al. 2004; Taheri et al. 2004). Furthermore, increased levels of the orexigenic hormone ghrelin has also been associated with sleep deprivation (Spiegel et al. 2004; Taheri et al. 2004). Also, epidemiologic studies consistently find that short sleep duration is associated with development of obesity in children and young adults (Nielsen et al. 2011). Although the epidemiologic evidence with regard to adults is less clear, self-reported reduction in sleep quality and duration has been associated with larger waist circumference, higher body mass index (BMI) and percentage of body fat, as well as risk of metabolic syndrome and diabetes in adults (Cappuccio et al. 2010; Xi et al. 2014).

The aim of the present study was to investigate whether residential exposure to road traffic and railway noise was associated with measures of adiposity and body composition among middle-aged adults, and to investigate whether certain groups are more vulnerable than others to road traffic noise.

## Methods

*Study population.* This cross-sectional study based on the Danish Diet, Cancer and Health cohort, which in the period 1993–1997 invited 160,725 persons 50–64 years of age to participate if they had no history of cancer (Tjønneland et al. 2007). In total, 57,053 chose to participate, yielding a response rate of 36% and representing 7% of the Danish population in this age group. At the time of enrollment, participants resided in either Aarhus or the greater Copenhagen area. In addition, study participants were born in Denmark and had no records of cancer before enrollment. Informed written consent was obtained from all participants, and the study was approved by local ethics committees.

*Anthropometry.* All anthropometric data was collected by trained laboratory technicians at the two centers (Copenhagen and Aarhus). Height was measured with participants standing without shoes and rounded to the nearest half centimeter. Weight was measured on a digital scale with participants in light clothing and rounded to the nearest 100 g. Waist circumference was measured at the narrowest part between the lower rib and the iliac crest and rounded to the nearest half centimeter. BMI was calculated from height and weight [weight (kilograms)/height^2^ (meters)] and analyzed both as a continuous and dichotomous variables (one with a cutoff at 25 kg/m^2^ and one with a cutoff at 30 kg/m^2^).

*Body composition.* For assessment of body composition bioelectrical impedance was measured using a BIA 101-F device (Akern/RJL), single frequency (50 Hz), with the participant in the supine position with legs ~ 45° apart and arms ~ 30° from the torso. Current electrodes were placed over the right metacarpals and metatarsals and sensing electrodes over the right wrist and ankle. The impedance measurements were used in sex-specific equations to calculate body fat mass:

BF(female) = 0.819BW – 0.279Ht^2^/R – 0.231Ht + 0.077age + 14.941 [1]

BF(male) = 0.755BW – 0.279Ht^2^/R – 0.231Ht + 0.077age + 14.941. [2]

The equations were developed and validated in a Danish population of similar age (Heitmann 1990) with BF (body fat in kilograms), BW (body weight in kilograms), Ht (height in centimeters), and R (resistance in ohms). Body fat mass index (BFMI) was calculated as BF divided by height squared, and lean body mass index (LBMI) was calculated as BFMI minus BMI (VanItallie et al. 1990).

*Exposure assessment.* Residential address history was collected for all cohort members for a period of 5 years preceding enrollment using the Danish civil registration system (Pedersen 2011). Road traffic noise exposure was calculated using SoundPLAN (http://www.soundplan.dk/), which implements the joint Nordic prediction method for road traffic noise (Bendtsen 1999). Equivalent noise levels were calculated for each address in a position on the most exposed facade of the building using the following input variables: point at which to estimate noise [geographical coordinate and height (floor) for each residential address], road links (information on annual average daily traffic, vehicle distribution, travel speed, and road type), and building polygons for all Danish buildings provided by the Danish Geodata Agency (http://www.gst.dk). We obtained traffic counts from a national road and traffic database (Jensen et al. 2009). This database is based on a number of different traffic data sources ranked as follows: *a*) traffic data (1995–1998) on municipal roads from 140 Danish municipalities, covering 97.5% of the addresses included in the present study; included roads typically have > 1,000 vehicles per day and are based on traffic counts as well as estimated/modeled numbers; *b*) traffic data from a central database covering all the major state and county roads; *c*) traffic data for 1995–2000 for all major roads in the greater Copenhagen area; *d*) smoothed traffic data for 1995 for all roads based on a simple method where estimated figures for distribution of traffic by road type and by urban–rural zone were applied to the road network and subsequently calibrated against known traffic data at county level. New roads were included in the calculations from the year they opened.

No information was available on noise barriers or road surfaces. Road traffic noise was calculated as the equivalent continuous A-weighted sound pressure level (L_Aeq_) at the most exposed facade of the dwelling at each address for the day (L_d_; 0700–1900 hours), evening (L_e_; 1900–2200 hours), and night (L_n_; 2200–0700 hours), and was expressed as L_den_ (den; day, evening, night). A 5- and 10-dB penalty was added to evening and night noise, respectively. Values < 42 dB were set to 42 dB because this is a realistic lower limit for ambient noise.

We calculated railway traffic noise exposure for all present and historical addresses within 300 m from a railway using SoundPLAN, implementing a Nordic calculation method for predicting noise propagation for railway traffic noise (NORD2000; http://www.soundplan.dk). The input variables for the noise model were point for noise estimation (geographical coordinate and height), railway links (information on annual average daily train lengths, train types, travel speed), and building polygons for all Danish buildings. All noise barriers along the railway were included in the model. Railway traffic noise was expressed as L_den_ at the most exposed facade of the dwelling and divided into four categories for the analyses (0–20; 20.1–50; 50.1–60; > 60 dB).

For the assessment of both road and railway traffic noise, the terrain was assumed flat, a reasonable assumption in Denmark; urban areas, roads, and areas with water were assumed to be hard surfaces, whereas all other areas were assumed acoustically porous.

The noise impact from all Danish airports and airfields was determined from information regarding noise zones calculated by local authorities from 1986 through 2003. The programs DANSIM (Danish Airport Noise Simulation Model) and INM3 (Integrated Noise Model), which meet joint Nordic criteria for air traffic noise calculations, were used (Liasjø and Granøien 1993). The curves for aircraft noise were transformed into digital maps and linked to each address by geocodes. A binary variable for aircraft noise was created from this information to indicate exposed and unexposed. Cut-off for exposure varied by airfield and level of detail of the obtained noise maps and was between 45 and 55 dB. Only 286 participants were exposed to aircraft noise at enrollment, and we did not have statistical power for an analysis of aircraft noise exposure.

*Air pollution.* We used the Danish AirGIS modeling system (dispersion model) to calculate the concentration of nitrogen oxides (NO_x_) for 5 years before enrollment at the addresses of the participants. The system has been described in detail elsewhere (Raaschou-Nielsen et al. 2011). Briefly, input data for the AirGIS system included traffic data for individual road links (same input data as described for the noise modeling), emission factors for the Danish car fleet, street and building geometry, building height, and meteorological data. The AirGIS system has been applied in several studies and validated, showing good performance for both short- and long-term averages (Ketzel et al. 2011).

*Covariates.* Information regarding smoking (never, former, and present as well as grams per day), alcohol consumption (grams per day), physical activity (recreational sport, hours per week), and diet (average intake per day of red meat, processed meat, poultry, bread, and soft drinks over the preceding 12 months) were obtained from detailed questionnaires completed and validated by interviewers at the time of enrollment. These variables were chosen based on their predictive values for development of obesity in a European meta-analysis including the Danish Diet, Cancer and Health cohort (Steffen et al. 2013).

Information on individual socioeconomic position (SEP) was obtained by linking the personal identification number of all cohort participants to the nationwide registers at Statistics Denmark, which have yearly information since 1980 from the taxation authorities and the Register for Education Statistics. For the year of enrollment, two socioeconomic indicators were defined: level of highest attained education [basic school/high school education (7–12 years of primary, secondary, and grammar school education), vocational training (10–12 years), and higher education (> 13 years)] and disposable income (household income after taxation and interest per person, adjusted for number of persons in the household and divided into tertiles based on the Danish background population). Municipality-level information was obtained based on enrollment address (or district for Copenhagen; 10 districts in total) classified as low, medium, or high based on municipality- or district-level information on education (proportion inhabitants with basic education), work market affiliation (proportion of inhabitants outside of the work market), and income (average income).

For analyses of effect modification we calculated a variable indicating whether participants had a comorbidity based on the Charlson comorbidity index. We created a dichotomous variable indicating whether the participant had a morbidity or not. Information regarding diseases was based on register information for hospitalizations from 1978 onward and included the following diseases: myocardial infarction, congestive heart failure, peripheral vascular disease, cerebrovascular disease, dementia, chronic pulmonary disease, connective tissue disease, ulcer disease, mild liver disease, diabetes type 1, diabetes type 2, hemiplagia, moderate-to-severe renal disease, diabetes with end-organ damage type 1 or type 2, moderate-to-severe liver disease and AIDS. The variable was dichotomized to express whether the participant had a comorbidity or not.

*Statistical analysis.* Analyses were based on the general linear model for continuous outcome measures and logistic regression for being overweight (BMI ≥ 25) or obese (BMI > 30). The assumption of linearity between dependent and continuous independent variables was examined visually and tested using linear spline models, and the variance of the residuals was assessed visually by plotting them against the predicted values. We found deviations from linearity for railway noise only in association with waist circumference and LBMI (for a visual assessment see restricted cubic splines, see Supplemental Material, Figure S1), and railway noise was therefore included in the analyses as a categorical variable. The variance of the residuals in all analyses was homogeneous (data not shown). Correlations between variables were assessed using Spearman rank correlation coefficients (*R*_s_). Exposures to road traffic noise and air pollution were modeled as time-weighted averages for 1 and 5 years preceding enrollment, taking into account the time of residence at each address. The procedures GLM and LOGISTIC in SAS version 9.3 (SAS Institute Inc.) were used for the statistical analyses. The graphical presentations of the association between road traffic noise and the adiposity measures were produced using restricted cubic spline in the rms library in the statistical software R 3.0.2 (R Core Team 2015).

Associations between 1- and 5-years mean exposure to road traffic noise and adiposity were analyzed in three models with increasing level of adjustment: model 1 adjusted for age and sex; model 2 further adjusted for SEP, railway and aircraft noise and model 3 added adjustment for lifestyle variables. Railway noise at enrollment was used as a proxy for prior exposure (the correlation between present and 5-year exposure to railway noise was *R*_s_ = 0.99) and analyzed as a categorical variable (0–20, 20.1–50, 50.1–60, and > 60 dB). Adjustment variables were chosen *a priori* using directed acyclic graphs (DAGs) as a visual and theoretical aid (Greenland et al. 1999), and the graphical tool DAGitty (http://www.dagitty.net) was used for constructing the DAGs (see Supplemental Material, Figure S2). The use of DAGs suggested a model adjusted for SEP for estimation of the total effect (no adjustment for potential causal intermediates) of traffic noise on adiposity (model 2); for the direct effect, a model adding adjustment for potential causal intermediates (alcohol consumption, smoking, diet, and physical activity) was suggested (model 3). We were interested primarily in the total effects of noise and place emphasized in model 2 when interpreting the results. Adjustment for age and sex was included in all models. To exclude the effect of competing noise sources, models 2 and 3 were adjusted for railway and aircraft noise. The analyses of railway noise exposure were adjusted for the same potential confounders as in the road traffic noise analyses, but also included continuous 5-year mean road traffic noise. Potential modification of the association between road traffic noise and adiposity measures by enrollment characteristics and age at diagnosis were evaluated by introducing interaction terms into the model, which were tested using the *F*-test statistic. Hypothesis testing for all analyses was based on two-tailed rejection regions, and an alpha level of 5% (*p*-value < 0.05) was applied to declare statistical significance.

## Results

From the cohort of 57,053 the following exclusions were made: 572 (1.00%) with a cancer diagnosis before enrollment; 162 (0.28%) due to missing, negative, or implausible values on outcome variables (height < 130 cm, BMI < 15, fat percentage < 2%); 1,970 (3.45%) due to incomplete exposure data; 1,893 (3.32%) due to missing values on adjustment variables. The final total study population was 52,456 participants.

Table 1 shows the distribution of covariates in the study population with regard to noise exposure in tertiles (the 5-year average road traffic noise is shown in Supplemental Material, Figure S3). The participants exposed to highest levels of road traffic noise (> 60 dB) more often had only basic education, lower income, and municipal SEP. They were more often smokers and did less recreational sport, as compared with the least exposed (< 54 dB).

**Table 1:** Characteristics of the population according to road traffic noise (*n* = 52,456).

Characteristic	Road traffic noise (L_den_) < 54 dB (*n* = 17,501)	Road traffic noise (L_den_) 54–60 (*n* = 17,475)	Road traffic noise (L_den_) > 60 dB (*n *= 17,480)
Age (years)	56.0 (50.7–64.1)	56.2 (50.7–64.2)	56.3 (50.8–64.3)
Sex (% men)	49.5	47.2	45.9
Highest attained education (%)
Basic	24.4	27.6	31.6
Vocational	44.9	45.9	45.5
Higher	30.7	26.6	22.9
SEP, municipal (%)^*a*^
Low	8.83	13.1	20.8
Medium	70.3	61.2	62.0
High	20.9	25.7	17.2
Disposable income (%)^*b*^
Low	6.25	9.3	14.2
Medium	17.4	21.2	25.7
High	76.4	69.5	60.1
Smoking (%)
Never	38.2	36.9	31.7
Former	29.6	28.2	26.8
Present	32.2	34.9	41.4
Smoking (g/day)^*c*^	15.0 (3.00–35.0)	15.0 (4.00–35.0)	16.0 (4.00–36.0)
Alcohol (g/day)	12.9 (0.92–61.1)	12.9 (0.71–62.9)	12.7 (0.51–69.0)
Red meat (g/day)	80.5 (34.4–165)	77.8 (31.8–164)	76.9 (29.6–166)
Processed meat (g/day)	25.3 (5.91–74.4)	24.2 (5.06–72.6)	24.4 (4.92–77.7)
Poultry (g/day)	18.3 (4.04–56.2)	18.2 (3.62–60.2)	17.2 (3.2–59.2)
Soft drinks (g/day)	7.96 (0.27–158)	7.96 (0.27–200)	7.17 (0.27–200)
Bread (g/day)	142 (55.3–270)	138 (52.1–267)	138 (49.8–264)
Recreational sport (hr/week) (%)
0	42.8	44.4	50.0
> 0–2	30.1	29.8	26.7
> 2	27.1	25.8	23.3
Comorbidity (% with a comorbidity)^*d*^	7.81	7.86	9.04
Waist circumference (cm)	89.0 (69.0–109)	89.0 (69.0–110)	88.0 (69.0–111)
BMI (kg/m^2^)	25.4 (20.5–32.8)	25.6 (20.5–33.5)	25.7 (20.4–33.9)
LBMI (kg/m^2^)	17.7 (15.0–21.0)	17.7 (15.1–21.2)	17.7 (15.1–21.4)
BFMI (kg/m^2^)	7.61 (4.20–13.5)	7.70 (4.19–13.9)	7.81 (4.13–14.2)
Road traffic noise (dB)	51.3 (46.7–53.8)	56.9 (54.3–60.0)	64.8 (60.8–73.0)
Railway noise (dB)^*e*^	50.8 (36.3–66.8)	50.6 (37.9–66.6)	51.5 (35.7–67.3)
Air pollution: NO_x_ (μg/m^3^)	16.8 (14.2–25.2)	20.8 (14.7–35.3)	36.6 (16.9–139)
Continuous variables are described using medians (5th–95th percentiles). No data were missing for any of the variables. ^***a***^Variable based on municipality level information. ^***b***^Tertiles based on the Danish background population. ^***c***^Among current and former smokers. ^***d***^Based on the Charlson Comorbidity Index (disease, no disease). ^***e***^Among railway noise–exposed participants.			

The correlation (*R*_s_) between 5-year road traffic noise exposure and 5-year air pollution (NO_x_) exposure was 0.70. One- and 5-year road traffic exposures were highly correlated (*R*_s_ = 0.97). Exposure to railway noise at the time of enrollment showed a correlation of 0.042 with road traffic noise exposure at enrollment. Measures of adiposity and body composition were generally highly correlated (*R*_s_ > 0.67) except the correlation between BFMI and LBMI, and between BFMI and waist circumference, for which *R*_s_ was 0.19 and 0.45, respectively.

**Figure 1:**
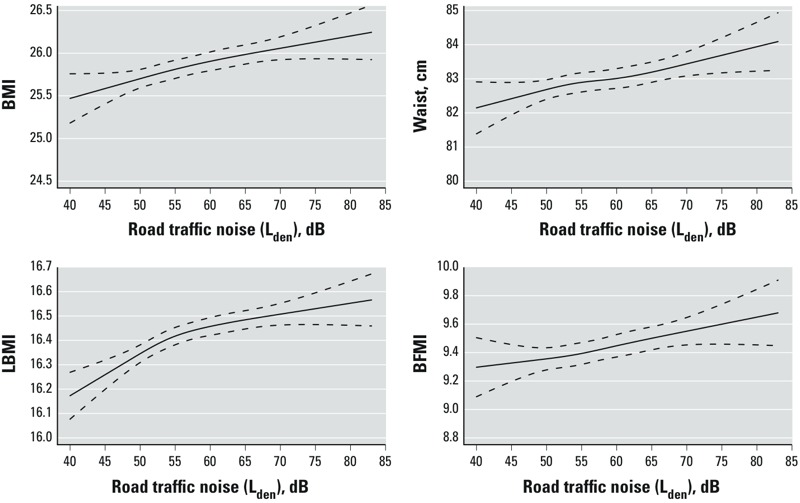
Associations between road traffic noise and measures of adiposity. Associations between 5-year exposure to residential road traffic noise and BMI, waist circumference, LBMI, and BFMI, adjusted for sex, age, disposable income, municipality SEP, education, railway noise, and aircraft noise (yes, no). Solid line indicates restricted cubic spline of the associations. Dotted lines indicate 95% CIs.

We found residential road traffic noise to be statistically significantly associated with all measures of body composition regardless of adjustment and exposure window (Table 2). For example, we estimated that for each 10-dB increase in 5-year exposure the waist circumference increased by 0.35 cm [95% confidence interval (CI): 0.21, 0.50] (model 2). The largest changes in estimates occurred from model 1 to model 2, where SEP and competing noise source variables were added. For example, the estimate for waist circumference changed from 0.39 cm per 10 dB (95% CI: 0.26, 0.53) to 0.30 cm per 10 dB (95% CI: 0.16, 0.44) for 1-year road traffic noise exposure. The estimates were slightly higher for the exposure period of 5 years compared with the exposure period of 1 year. For example, the estimate for BMI in model 2 for 1-year exposure was 0.16 points per 10 dB (95% CI: 0.11, 0.21), whereas it was 0.18 points per 10 dB (95% CI: 0.12, 0.23) for 5-year exposure in model 2. Adjustment for lifestyle factors (model 3) did not alter the estimates much. Further adjustment for air pollution (NO_x_) resulted in only minor changes in estimates (results not shown). When we modeled road traffic noise using restricted cubic splines, exposure–response relationships were positive and increased monotonically over the entire exposure range, with was no apparent threshold (Figure 1). For further analyses of BMI as a dichotomous outcome, the odds ratio (OR) for being overweight/obese (BMI ≥ 25 compared with < 25) with a 10-dB increase in road traffic noise was 1.10 (95% CI: 1.07, 1.14) for model 2 and 1.11 (95% CI: 1.08, 1.15) for model 3. Similarly, the OR for being obese (BMI > 30 compared with ≤ 30) was 1.09 (95% CI: 1.05, 1.14) based on models 2 and 3.

**Table 2:** Linear associations between 1- and 5-years mean residential exposure to road traffic noise (Lden) and measures of adiposity [difference per 10 dB (95% CI)].

Per 10-dB higher road traffic noise exposure	Model 1^*a*^	Model 2^*b*^	Model 3^*c*^
1 year preceding enrollment
BMI (kg/m^2^)	0.23 (0.18, 0.28)	0.16 (0.11, 0.21)	0.17 (0.12, 0.22)
Waist circumference (cm)	0.39 (0.26, 0.53)	0.30 (0.16, 0.44)	0.25 (0.11, 0.39)
LBMI (kg/m^2^)	0.15 (0.13, 0.16)	0.079 (0.061, 0.097)	0.084 (0.066, 0.10)
BFMI (kg/m^2^)	0.089 (0.089, 0.13)	0.081 (0.042, 0.12)	0.083 (0.045, 0.12)
5 years preceding enrollment
BMI (kg/m^2^)	0.25 (0.20, 0.30)	0.18 (0.12, 0.23)	0.19 (0.13, 0.24)
Waist circumference (cm)	0.44 (0.30, 0.58)	0.35 (0.21, 0.50)	0.30 (0.16, 0.45)
LBMI (kg/m^2^)	0.15 (0.13, 0.17)	0.085 (0.066, 0.10)	0.090 (0.072, 0.11)
BFMI (kg/m^2^)	0.10 (0.066, 0.14)	0.097 (0.057, 0.14)	0.10 (0.061, 0.14)
^***a***^Adjusted for sex and age. ^***b***^Model 1 plus adjustment for disposable income, municipality SEP, education, railway noise and aircraft noise (yes, no). ^***c***^Model 2 plus adjustment for smoking (yes, no), tobacco (g/day), alcohol (yes, no), alcohol (g/day), red meat (g/day), processed meat (g/day), poultry (g/day), bread (g/day), soft drinks (g/day), recreational sport (yes, no), and recreational sport (hours/week).			

Table 3 shows the associations between exposure to residential railway noise at enrollment in three categories and measures of adiposity and body composition. The group with the highest exposure (> 60 dB) had statistically significant larger mean waist circumference, mean BMI, and mean LBMI in all models. For example, mean waist circumference was 0.71 cm (95% CI: 0.23, 1.19) wider, mean BMI was 0.19 points (95% CI: 0.0072, 0.37) higher, and mean LBMI was 0.063 points (95% CI: 0.0016, 0.12) higher among those exposed to more than 60 dB railway noise at enrollment compared with the unexposed (model 2). The associations between railway noise and BFMI were not statistically significant in all models, but showed similar tendencies of increased estimates for the highly exposed.

**Table 3:** Associations between average yearly residential exposure to railway traffic noise (L_den_) at the time of enrollment and measures of adiposity.

Exposure	*n*	Model 1^*a*^ estimate (95% CI)	Model 2^*b*^ estimate (95% CI)	Model 3^*c*^ estimate (95% CI)
BMI (kg/m^2^)
0–20 dB	42,747	Reference	Reference	Reference
20.1–50 dB	4,482	0.042 (–0.082, 0.17)	0.099 (–0.025, 0.22)	0.12 (0.0030, 0.25)
50.1–60 dB	3,308	0.053 (–0.090, 0.20)	0.024 (–0.12, 0.17)	0.031 (–0.11, 0.17)
> 60 dB	1,919	0.28 (0.10, 0.47)	0.19 (0.0072, 0.37)	0.18 (0.0034, 0.36)
Waist circumference (cm)
0–20 dB	42,747	Reference	Reference	Reference
20.1–50 dB	4,482	0.025 (–0.30, 0.35)	0.22 (–0.10, 0.55)	0.27 (–0.054, 0.59)
50.1–60 dB	3,308	0.13 (–0.25, 0.50)	0.12 (–0.25, 0.49)	0.15 (–0.22, 0.51)
> 60 dB	1,919	0.89 (0.40, 1.37)	0.71 (0.23, 1.19)	0.62 (0.14, 1.09)
LBMI (kg/m^2^)
0–20 dB	42,747	Reference	Reference	Reference
20.1–50 dB	4,482	0.060 (0.019, 0.10)	0.048 (0.0063, 0.089)	0.057 (0, 016, 0.097)
50.1–60 dB	3,308	0.060 (0.0090, 0.10)	0.013 (–0.034, 0.060)	0.015 (–0.031, 0.062)
> 60 dB	1,919	0.12 (0.054, 0.18)	0.063 (0.0016, 0.12)	0.063 (0.0033, 0.12)
BFMI (kg/m^2^)
0–20 dB	42,747	Reference	Reference	Reference
20.1–50 dB	4,482	–0.018 (–0.11, 0.071)	0.051 (–0.038, 0.14)	0.068 (–0.019, 0.15)
50.1–60 dB	3,308	–0.0031 (–0.11, 0.10)	0.011 –0.091, 0.11)	0.016 (–0.084, 0.12)
> 60 dB	1,919	0.17 (0.033, 0.30)	0.13 (–0.0041, 0.26)	0.12 (–0.0097, 0.25)
^***a***^Adjusted for sex and age. ^***b***^Model 1 plus adjustment for disposable income, municipality SEP, education, road traffic noise and aircraft noise (yes, no). ^***c***^Model 2 plus adjustment for smoking (yes, no), tobacco (g/day), alcohol (yes, no), alcohol (g/day), red meat (g/day), processed meat (g/day), poultry (g/day), bread (g/day), soft drinks (g/day), recreational sport (yes, no), and recreational sport (hours/week).				

For LBMI, we found a significant effect modification by age (*p* = 0.018), with higher estimates for the association between road traffic noise and LBMI among participants < 56 years of age (0.10; 95% CI: 0.075, 0.13) compared with older participants (0.016; 95% CI: 0.033, 0.089) (Table 4). We also found evidence of effect modification by comorbidity, with no association between road traffic noise and BMI and BFMI among participants with comorbidity, and a positive association among those without comorbidity (e.g., for BMI, 0.017; 95% CI: –0.17, 0.20; and 0.21; 95% CI: 0.14, 0.28, respectively, interaction *p*-value 0.04). Comorbidity was not a statistically significant effect modifier for other associations. We also found effect modification of the association between road traffic noise and adiposity measures by railway noise for BMI, waist circumference, and BFMI, with higher estimates for railway noise–exposed individuals. However, the associations were weakest for the middle stratum (railway noise: 20–60 dB), and only for waist circumference did the CIs for the lowest (0–19.9 dB) and highest strata (> 60 dB) not overlap. We found no effect modification for other covariates (sex, income, and education).

**Table 4:** Modification of associations between 5-year road traffic noise exposure (L_den_) and measures of adiposity by enrollment characteristics in model 2 [difference per 10 dB (95% CI)].

Potential effect modifier/covariate level	*n*	BMI (kg/m^2^)^*a*^	Waist circumference (cm)^*a*^	LBMI (kg/m^2^)^*a*^	BFMI (kg/m^2^)^*a*^
Sex
Male	24,916	0.19 (0.10, 0.27)	0.40 (0.18, 0.63)	0.10 (0.067, 0.12)	0.093 (0.031, 0.115)
Female	27,540	0.20 (0.12, 0.28)	0.30 (0.084, 0.52)	0.07 (0.043, 0.10)	0.13 (0.071, 0.19)
*p*‑Value		0.81	0.47	0.16	0.32
Income tertiles^*b*^
Highest	36,005	0.22 (0.14, 0.29)	0.36 (0.073, 0.66)	0.094 (0.074, 0.12)	0.12 (0.070, 0.18)
Middle	11,244	0.19 (0.067, 0.31)	0.40 (0.17, 0.63)	0.059 (0.019, 0.10)	0.13 (0.041, 0.21)
Lowest	5,207	0.034 (–0.14, 0.20)	0.26 (–0.024, 0.54)	0.040 (–0.017, 0.10)	–0.0059 (–0.13, 0.12)
*p*‑Value		0.11	0.36	0.075	0.11
Education
Higher	14,020	0.20 (0.089, 0.31)	0.30 (0.014, 0.58)	0.086 (0.049, 0.12)	0.11 (0.034, 0.19)
Vocational	23,827	0.21 (0.13, 0.30)	0.35 (0.13, 0.58)	0.092 (0.063, 0.12)	0.12 (0.057, 0.18)
Basic	14,609	0.16 (0.055, 0.27)	0.25 (–0.025, 0.53)	0.061 (0.026, 0.097)	0.10 (0.024, 0.18)
*p*‑Value		0.73	0.70	0.33	0.92
Age (years)
≤ 56.2	26,228	0.22 (0.13, 0.30)	0.39 (0.17, 0.61)	0.10 (0.075, 0.13)	0.12 (0.054, 0.18)
> 56.2	26,228	0.17 (0.087, 0.26)	0.31 (0.090, 0.53)	0.061 (0.033, 0.089)	0.11 (0.050, 0.17)
*p*‑Value		0.38	0.59	0.018	0.90
Comorbidity^*c*^
Yes	4,320	0.017 (–0.17, 0.20)	–0.0086 (–0.49, 0.47)	0.044 (–0.017, 0.11)	–0.015 (–0.16, 0.10)
No	48,136	0.21 (0.14, 0.28)	0.37 (0.19, 0.55)	0.085 (0.063, 0.10)	0.12 (0.075, 0.17)
*p*‑Value		0.042	0.13	0.19	0.027
Railway noise (dB)
0–19.9	42,893	0.20 (0.14, 0.26)	0.40 (0.24, 0.56)	0.090 (0.070, 0.11)	0.11 (0.070, 0.49)
20–60	7,765	0.00057 (–0.14, 0.14)	–0.11 (–0.48, 0.26)	0.042 (–0.0046, 0.089)	–0.042 (–0.14, 0.059)
> 60	1,798	0.42 (0.13, 0.70)	1.33 (0.58, 2.08)	0.13 (0.039, 0.23)	0.28 (0.077, 0.49)
*p*‑Value		0.0079	0.0014	0.10	0.0039
*p*‑Values represent an *F*-test for the interaction term, the null hypothesis being no interaction. ^***a***^Adjusted for sex, age, disposable income, municipality SEP, education, railway noise and aircraft noise (yes, no). ^***b***^Tertiles based on the Danish background population. ^***c***^Based on the Charlson Comorbidity Index (disease, no disease).					

## Discussion

In this large cross-sectional study of middle-aged Danish men and women, we found consistent and statistically significant positive associations between road traffic noise and waist circumference, BMI, BFMI, and LBMI. Road traffic noise was also associated with a higher prevalence of being overweight and obese. Furthermore, we estimated statistically significant positive associations between exposure to railway noise > 60 dB (vs. 0–20 dB) and BMI, waist circumference, and LBMI, and a nonsignificant positive association with BFMI. In addition, we found a significant effect modification by railway noise with a stronger association between road traffic noise and BMI, waist circumference, and BFMI among persons exposed to > 60 dB of railway noise. However, the observed associations did not increase with increasing railway noise, because associations were weakest for the middle stratum (railway noise, 20–60 dB).

Traffic noise has been associated with both cardiovascular disease (Babisch 2008; Sørensen et al. 2011) and diabetes (Sørensen et al. 2013), diseases for which adiposity is a major risk factor (Haslam and James 2005). A recent study addressed the associations between noise exposure and adiposity measures, finding a positive association between aircraft noise and increase in waist circumference (Eriksson et al. 2014). Taken together with the results of the current study, the evidence suggests that noise-related effects on adiposity may contribute to associations between traffic noise and major diseases. We found a clear exposure–response relationship between exposure to road traffic noise and all measures of body composition, with no indications of any lower threshold limit. We observe a positive association for LBMI. This is to be expected because the metabolically active lean body mass (LBM) increases with increasing body fat mass (BFM) (VanItallie et al. 1990), potentially because larger BFM requires larger organs and musculoskeletal system. LBMI and BFMI were positively correlated in this study (*R*_s_ = 0.75 among women and *R*_s_ = 0.70) as well as in other studies (Bosy-Westphal and Müller 2015). Some studies on road traffic noise and risk for cardiovascular disease have indicated a lower threshold limit of 55–60 dB (Babisch 2008; Sørensen et al. 2011), though a study on diabetes and a study on myocardial infarction similar to the present study indicated no lower threshold (Sørensen et al. 2012, 2013). More studies are needed to determine whether there is a lower threshold limit for the adverse effects of road traffic noise.

Railway and road traffic noise were very weakly correlated in this study (*R*_s_ = 0.04), which suggests that associations of the two exposures with adiposity were unlikely to be mutually confounded. For railway noise we found an association with adiposity only among participants exposed to > 60 dB.

Our results for road traffic noise were statistically significant regardless of adjustment level. The largest change in estimates occurred when we included adjustment for SEP variables and competing noise sources. Considering the proposed mechanisms of action—namely, stress and disturbance of sleep—several lifestyle factors such as diet and physical activity may be considered as being on the pathway between noise exposure and development of adiposity. To examine the effect of lifestyle on the association, we adjusted for diet, smoking, alcohol consumption, and physical activity. This adjustment did not alter the estimates markedly, and although the necessary assumptions for mediation analyses are not met, it may indicate that noise does not act primarily through these factors. However, information on lifestyle was obtained by questionnaires at the same point in time as the measurement of adiposity markers and are therefore possibly subject to recall bias, as possible associations between lifestyle and adiposity may be caused by reverse causality.

Road traffic noise is correlated with ambient air pollution; air pollution has been suggested to be associated with the development of obesity in mice (Xu et al. 2010); and recent results in humans suggest a possible association between air pollution and obesity among children (McConnell et al. 2015). To investigate potential confounding by air pollution, we adjusted for modeled NO_x_ (data not shown). We found that exposure to road traffic noise was significantly associated with adiposity both before and after adjustment for air pollution, suggesting an independent effect of road traffic noise.

We examined potential effect modification of sex, SEP (income and education), age, comorbidity, and railway noise. The results indicated that the association between road traffic noise and LBMI was weaker among those above the median age (56.2 years) compared with younger participants. However, this pattern was not present for other outcomes. Road traffic noise was not associated with BMI or BFMI among participants classified as having comorbidity based on the Charlson comorbidity index. Associations with waist circumference and LBMI also were close to the null for this group, though the *p*-value for effect modification was not statistically significant. This suggests that effects of road traffic noise on adiposity may be negligible compared with direct and indirect effects of illness on body composition, though noncausal explanations also are possible. Sex and SEP (education or income) did not appear to modify associations between road traffic noise and the adiposity measures.

We found associations between road traffic noise and BMI, waist circumference, and BFMI to be strongest among participants exposed to > 60 dB railway noise. However, the middle, not the lower, stratum consistently displayed the weakest associations. Furthermore, only the CIs for waist circumference did not overlap, indicating that there may not be a difference between the lowest and highest stratum for BMI and BFMI.

Major strengths of this study were its size and the availability of detailed information regarding diet, alcohol consumption, smoking, and recreational physical activity; the objective measurements of weight, height, and bioimpedance by trained staff; and access to individual-level register data on SEP, residential address history for noise assessment, and air pollution data for all study subjects. Exposure data are based on modeled levels of road and railway noise at addresses and therefore largely independent of information bias.

Some limitations deserve consideration. First, the participation rate in the study was low (36%), which can introduce bias if nonparticipation is associated with both exposure and outcome. The participants in the Danish Diet, Cancer and Health cohort are known to be of higher SEP than nonparticipants (Tjønneland et al. 2007). However, during the first years of follow-up (baseline to 31 December 2002) they did not develop cancer (all malignancies except non-melanoma skin cancer) at a different rate than the background population. Futhermore, their questionnaire responses were similar to those in studies with higher participation rates (Levnedsmiddelstyrelsen 1996). Taken together, these results may suggest that the study population is similar to the background population (Tjønneland et al. 2007). Second, this is a cross-sectional study, so the temporal relation between the exposures and outcomes is uncertain. Third, the largest changes in effect estimates were observed when we added adjustment for SEP, and the possibility of residual confounding cannot be excluded. Fourth, traffic noise is modeled and yields an exposure estimate at the most exposed facade of the residence, whereas a more relevant exposure may be at the bedroom facade when considering sleep disturbance as a mechanism. Finally, we did not have information on noise barriers for road traffic noise or individual-level noise modifiers such as building materials, and the noise modeling is therefore invariably associated with some degree of uncertainty leading to misclassification of exposure.

Although noise exposure modeling has some limitations, there are reasons to prefer modeling to measuring. Most important, modeling makes large-scale exposure assessment possible; a cohort study of this size would not be feasible using traditional measuring methods. Furthermore, measuring is usually performed during a relatively short period of time, and several factors influence measuring in the short term that may yield a flawed exposure estimate. First, the level of traffic noise varies over time due to the movement of particular vehicles relative to the observer. Second, the busy rush hours and quiet night period cause a characteristic diurnal variation. Last, the weather strongly influences the propagation of traffic noise and thereby the noise levels. The scale used to characterize traffic noise is the long-term average noise level, assuming standardized weather conditions. This quantity is difficult to measure directly. During the last four decades still more accurate and reliable prediction methods for traffic noise have been developed, and a Swedish report found that modeled values differed from the measured values by an average of only 0.3 dB for the Nordic Prediction Method (Ström et al. 1997).

## Conclusions

In conclusion, this study based on data on middle-aged Danish adults from the 1990s adds to the mounting evidence that road traffic noise may have adverse health effects. We found statistically significant positive associations between road traffic noise and different markers of adiposity. We also found nonlinear positive association between railway noise and BMI, waist circumference, and LBMI for railway noise exposure > 60 dB. Because this area of research is relatively new, further research is needed to confirm the findings of this study.

## Supplemental Material

(461 KB) PDF
