# Supplementary material for: Road Traffic and Railway Noise Exposures and Adiposity in Adults: A Cross-Sectional Analysis of the Danish Diet, Cancer, and Health Cohort
Source: Environ Health Perspect. 2015 Aug 4;124(3):329–35. doi: 10.1289/ehp.1409052 (PMC4786981; doi:10.1289/ehp.1409052)
Supplement: (461 KB) PDF [file ehp.1409052.s001.acco.pdf]

Environ Health Perspect
DOI: 10.1289/ehp.1409052

Note to Readers: *EHP* strives to ensure that all journal content is accessible to all readers. However, some figures and Supplemental Material published in *EHP* articles may not conform to 508 standards due to the complexity of the information being presented. If you need assistance accessing journal content, please contact ehp508@niehs.nih.gov. Our staff will work with you to assess and meet your accessibility needs within 3 working days.

Supplemental Material

Road Traffic and Railway Noise Exposures and Adiposity in Adults: A Cross-Sectional Analysis of the Danish Diet, Cancer, and Health Cohort

Jeppe Schultz Christensen, Ole Raaschou-Nielsen, Anne Tjønneland, Kim Overvad, Rikke B. Nordsborg, Matthias Ketzel, Thorkild IA Sørensen, and Mette Sørensen

Table of Contents

Figure S1. Associations between residential railway noise exposure at enrolment and BMI, waist circumference, LBMI and BFMI, adjusted for sex, age, disposable income, municipality SEP, education, road traffic noise and aircraft noise (yes, no). Solid line: restricted cubic spline of the associations. Dotted lines: 95% confidence limits.

Figure S2. Directed acyclic graph of the association between traffic noise and adiposity.

Figure S3. Distribution of five year average road traffic noise prior to enrollment in the Danish Cancer and Health cohort.

Environ Health Perspect
DOI: 10.1289/ehp.1409052

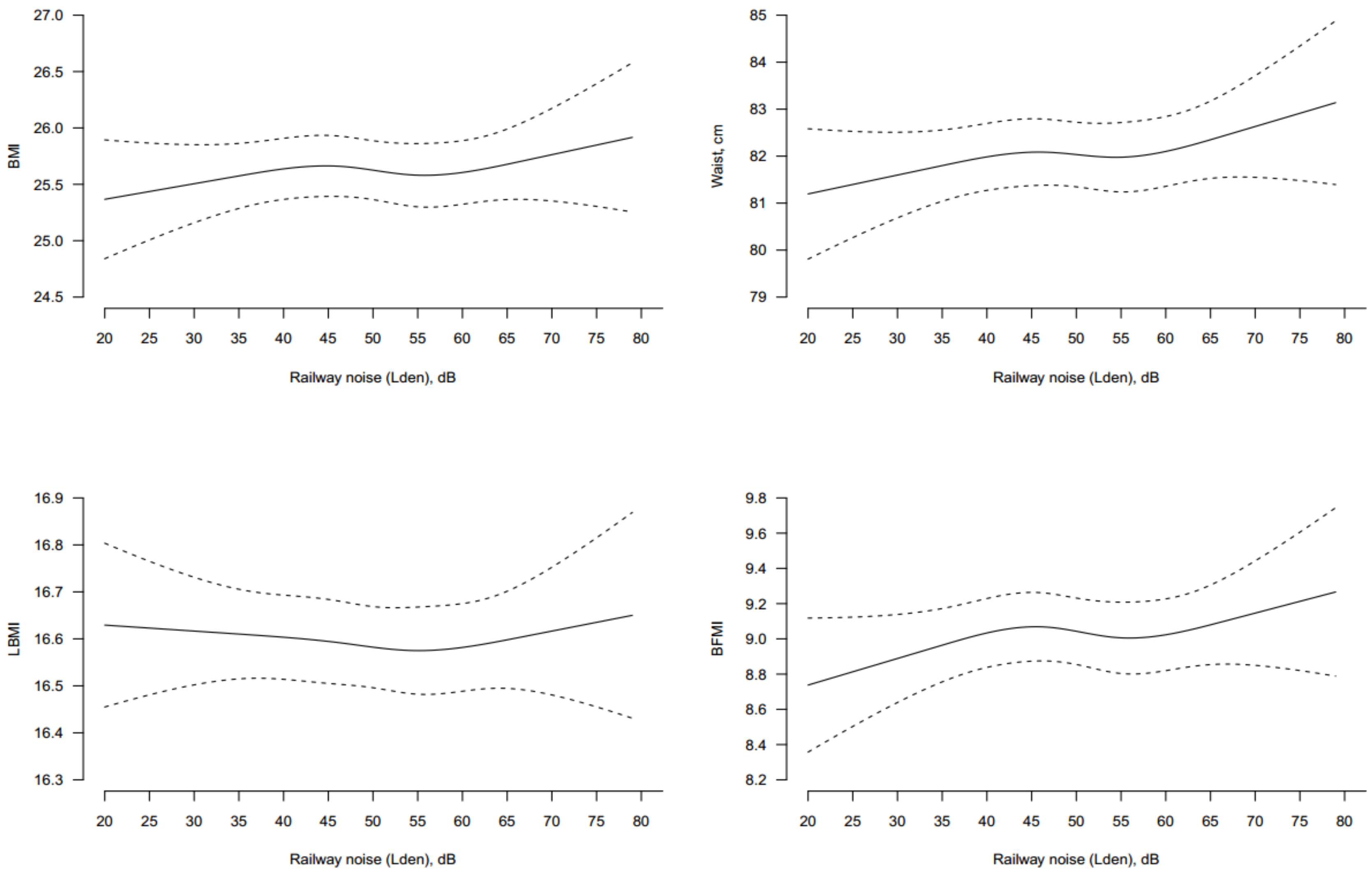

Figure S1. Associations between residential railway noise exposure at enrolment and BMI, waist circumference, LBMI and BFMI, adjusted for sex, age, disposable income, municipality SEP, education, road traffic noise and aircraft noise (yes, no). Solid line: restricted cubic spline of the associations. Dotted lines: 95% confidence limits.

DOI: 10.1289/ehp.1409052

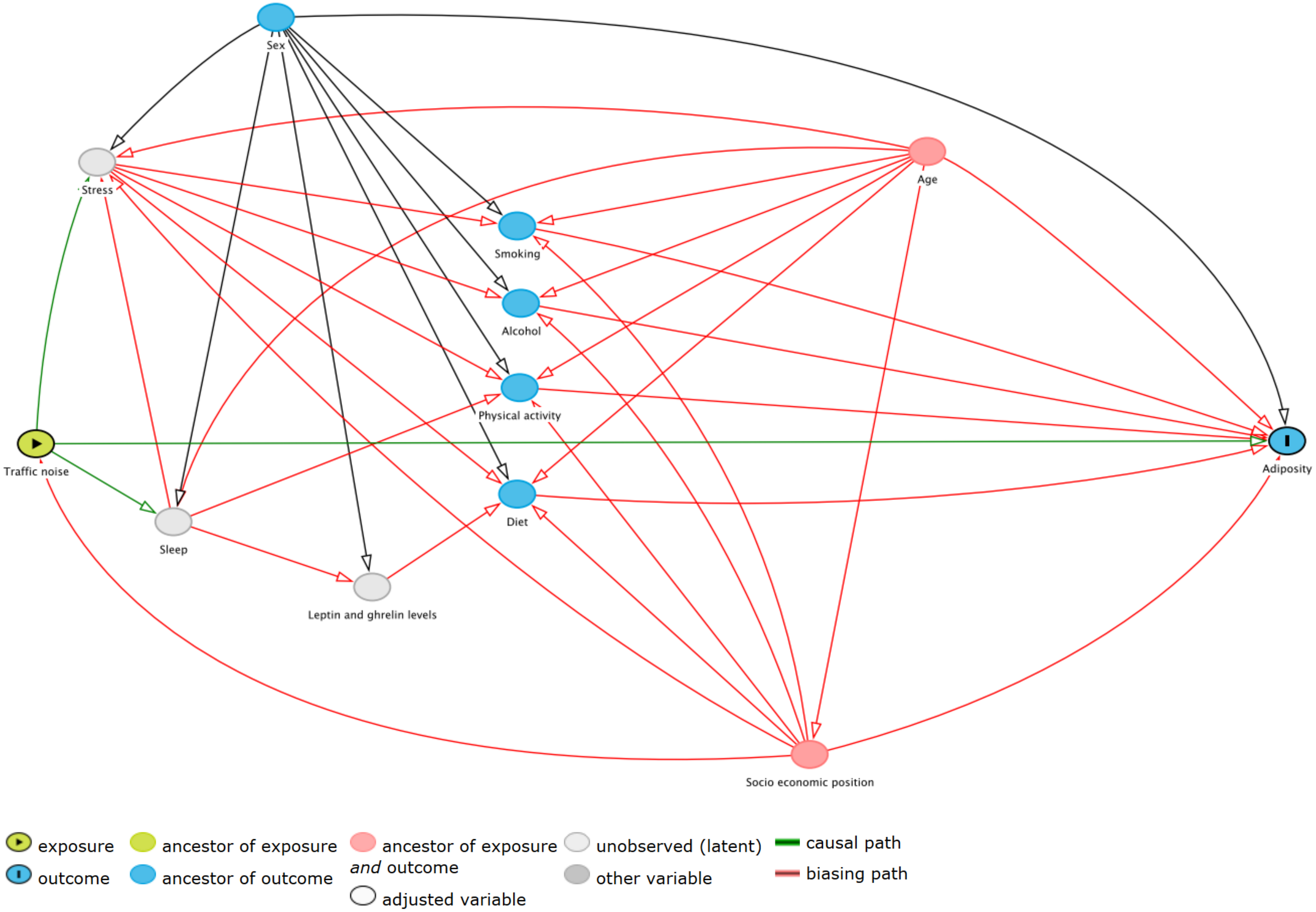

Figure S2. Directed acyclic graph of the association between traffic noise and adiposity.

DOI: 10.1289/ehp.1409052

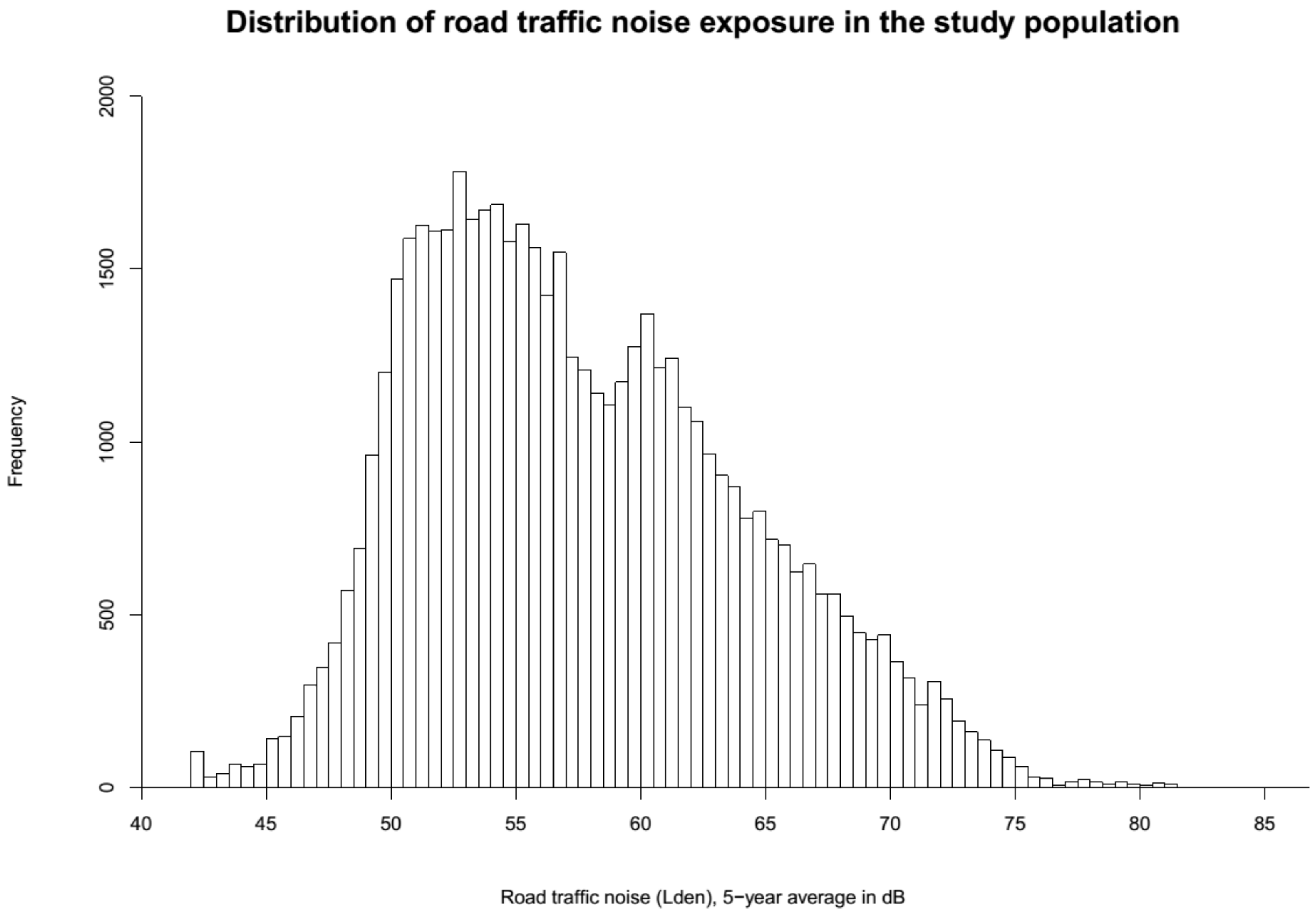

Figure S3. Distribution of five year average road traffic noise prior to enrollment in the Danish Cancer and Health cohort.
